# Loneliness, worries, anxiety, and precautionary behaviours in response to the COVID-19 pandemic: A longitudinal analysis of 200,000 Western and Northern Europeans

**DOI:** 10.1016/j.lanepe.2020.100020

**Published:** 2021-01-02

**Authors:** Tibor V. Varga, Feifei Bu, Agnete S. Dissing, Leonie K. Elsenburg, Joel J. Herranz Bustamante, Joane Matta, Sander K.R. van Zon, Sandra Brouwer, Ute Bültmann, Daisy Fancourt, Klaus Hoeyer, Marcel Goldberg, Maria Melchior, Katrine Strandberg-Larsen, Marie Zins, Amy Clotworthy, Naja H. Rod

**Affiliations:** a Section of Epidemiology, Department of Public Health, University of Copenhagen, Copenhagen, Denmark; b Department of Behavioural Science and Health, University College London, 1-19 Torrington Place, London, WC1E 7HB, UK; c Sorbonne Université, INSERM, Institut Pierre Louis d'Epidémiologie et de Santé Publique (IPLESP), Equipe de Recherche en Epidémiologie Sociale (ERES), Paris, France; d Inserm, Population-based Epidemiological Cohorts Unit, UMS 011, 94800 Villejuif, France; e Department of Health Sciences, Community and Occupational Medicine, University of Groningen, University Medical Center Groningen, Groningen, The Netherlands; f Section for Health Services Research, Department of Public Health, University of Copenhagen, Copenhagen, Denmark; g Faculté de Médecine, Université de Paris, 75006 Paris

**Keywords:** COVID-19, Pandemic, Public health, Global health, Lockdown, Governmental interventions, Loneliness, Anxiety, Worries, Precautions

## Abstract

**Background:**

In response to the COVID-19 pandemic, governments around the world instituted various public-health measures. Our project aimed to highlight the most significant similarities and differences in key mental-health indicators between four Western and Northern European countries, and identify the population subgroups with the poorest mental-health outcomes during the first months of the pandemic.

**Methods:**

We analysed time-series survey data of 205,084 individuals from seven studies from Denmark, France, the Netherlands, and the UK to assess the impact of the pandemic and associated lockdowns. All analyses focused on the initial lockdown phase (March–July 2020). The main outcomes were loneliness, anxiety, and COVID-19-related worries and precautionary behaviours.

**Findings:**

COVID-19-related worries were consistently high in each country but decreased during the gradual reopening phases. While only 7% of the respondents reported high levels of loneliness in the Netherlands, percentages were higher in the rest of the three countries (13–18%). In all four countries, younger individuals and individuals with a history of mental illness expressed the highest levels of loneliness.

**Interpretation:**

The pandemic and associated country lockdowns had a major impact on the mental health of populations, and certain subgroups should be closely followed to prevent negative long-term consequences. Younger individuals and individuals with a history of mental illness would benefit from tailored public-health interventions to prevent or counteract the negative effects of the pandemic. Individuals across Western and Northern Europe have thus far responded in psychologically similar ways despite differences in government approaches to the pandemic.

**Funding:**

See the Funding section.

**Research in context**

**Evidence before this study**

In response to the COVID-19 pandemic, national governments around the world responded to the crisis with specific public-health interventions, such as quarantining citizens and instituting curfews. Several previous reports indicate that the COVID-19 pandemic and its associated preventive measures have had varying impacts on the mental health of different population subgroups, with young adults and those with previous chronic diseases reporting poorer outcomes. Psychological distress, loneliness, and anxiety have the potential to develop into long-term and severe mental illness with significant individual and socioeconomic consequences.

**Added value of this study**

Using a large cross-national sample of >200,000 individuals, our analysis indicates that across the four Western and Northern European countries investigated, individuals have responded in psychologically similar ways to the pandemic and its associated preventive measures despite differences in government approaches. In addition, we observed consistency in key mental-health indicators across the four countries; however, respondents with previously diagnosed mental illness and younger respondents reported poorer mental health during the first months of the COVID-19 pandemic.

**Implications of all the available evidence**

Combined evidence from previous studies and ours indicate that younger individuals and people with a history of mental illness experienced higher levels of loneliness compared to other subgroups during the first four months of the societal lockdowns related to the COVID-19 pandemic. As such, we recommend further explorations of the effects of specific interventions with an aim to alleviate poor mental-health outcomes in these subgroups. As the pandemic continues, rather than recommending one-size-fits-all public-health interventions, future governmental measures should include targeted strategies for subgroups with potentially different needs in order to decrease the risk of serious long-term health consequences. Our study is the first to show consistency between mental-health landscapes (defined as holistic overviews of important mental-health outcomes) and subgroup trends across four European countries, despite their varying governmental interventions, lockdown strategies, and general stringency. These findings suggest that a coordinated international strategy and increased collaboration would be effective in counteracting the negative impacts of the pandemic and its related lockdowns on mental health.

Alt-text: Unlabelled box

## 1 Introduction

With the purpose of containing the spread of the SARS-CoV-2 virus in March 2020, many governments decided to implement strict public-health measures, such as quarantining citizens and instituting curfews. The most common precautions recommended to the general population were to increase hand hygiene, remain physically distant from others, self-isolate, avoid crowded public places, avoid travel, and wear face-masks.[1], [2], [3] Many European governments, such as those of Denmark, France, the Netherlands, and the United Kingdom, also decided to close borders and temporarily close businesses, schools, and public activities to prevent the virus's spread and reduce strain on the healthcare sector.[4] The governments of some countries, including those of France and the United Kingdom, imposed curfews and even stricter public-health measures to contain the spread of infection.

For many people, the COVID-19 pandemic prompted feelings of social isolation, uncertainty, depression, stress reaction, generalised anxiety, and fear of the virus.[1,2] As depression and anxiety have been linked to COVID-19-related news in smaller samples, [5] it is likely that certain political interventions and public announcements during the first months of the pandemic directly impacted overall mental-health outcomes; e.g., levels of loneliness, feelings of isolation, worries, and anxiety. As each country addressed the pandemic in its own specific manner and pace, it is possible that individual perceptions and reactions across countries varied. Thus far, there is a lack of larger-scale, longitudinal, cross-national comparative studies and comparisons on mental-health indicators.

In this study, we conducted a cross-national comparison between seven longitudinal studies from four European countries in order to investigate whether mental-health outcomes varied in response to different governmental strategies. There is a growing literature that investigates the psychological and mental-health effects of the COVID-19 pandemic and its associated societal lockdowns on various population subgroups, but few studies have used large population samples representative of the general population [6] and involving comparisons between various groups of individuals. Previous research indicates that societal lockdowns and periods of social isolation may contribute to increased levels of loneliness, worry, depression, and anxiety in many people; as such, the mental health of populations during the COVID-19 pandemic has emerged as a serious public-health concern.[7], [8], [9]

In rapid response to the COVID-19 outbreak, a number of Western and Northern European cohorts created and harmonised a targeted survey to examine the pandemic's effects on common mental-health indicators in general populations. Since then, there has been increased collaboration between these teams to undertake cross-national comparisons of the mental-health impact of the COVID-19 pandemic; these have been facilitated by international networks such as the COVID-MINDS Network, which focuses on the global mental-health impact of the pandemic [www.covidminds.org]. The study presented here documents mental-health outcomes of populations during the first four months of the COVID-19 pandemic, drawing upon data from seven cohorts across four Western and Northern European countries. We overlaid these data with timelines of country-wide government interventions. The March–July timeframe was selected to assess the impact of the immediate phase of the lockdown in each country. Furthermore, we assessed whether levels of loneliness, worries, anxiety, and specific precautionary behavioural changes in response to the lockdown differed between these countries.

## 2 Methods

### 2.1 Participating cohorts

**Fig. 1:**
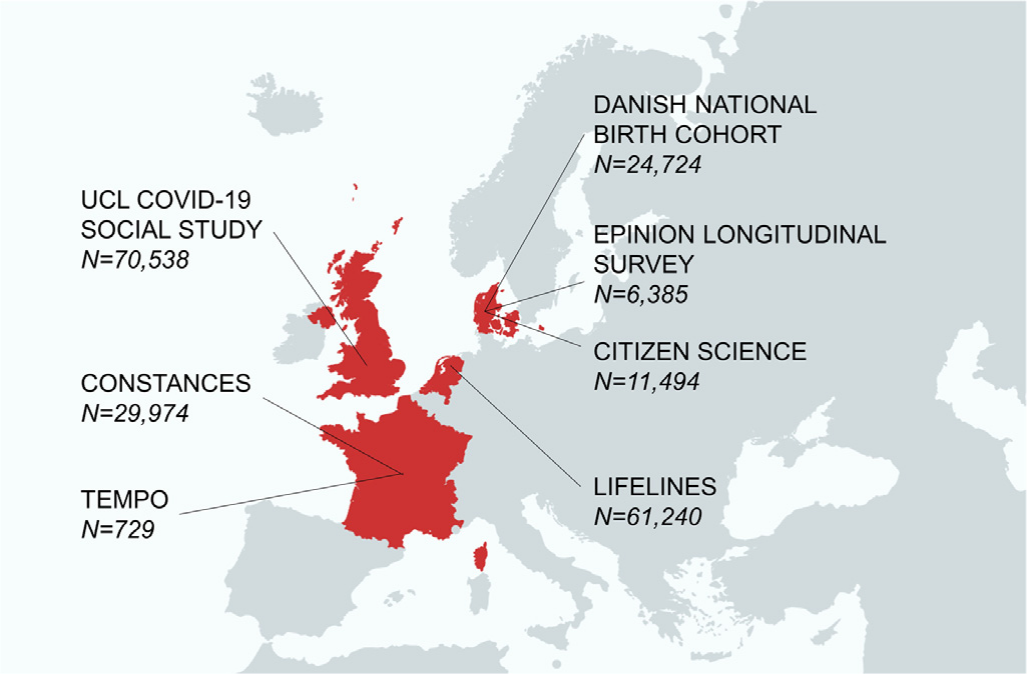
Participating countries and cohorts (N=205,084).

The seven participating cohorts from four countries are presented in Fig. 1. The participating cohorts are described in Supplemental Text 1.

A quantitative survey was harmonised and distributed to multiple cohorts in the COVID-MINDS Network. Covering a range of items, the survey included questions on basic sociodemographic measures (age, gender, level of education, cohabitation and occupational status during the pandemic); chronic physical and mental disorders; perceived loneliness; COVID-19-related mental-health indicators; and COVID-19-related precautions and worries. From Denmark, the Danish National Birth Cohort (DNBC) (*N* = 24,724), the Citizen Science cohort (*N* = 11,494), and the Epinion time-series data (*N* = 6,385) were analysed.[3] From France, the Constances (*N* = 29,974) and TEMPO (*N* = 729) cohorts were analysed.[10,11] The individuals from the Constances cohort were also members of the SAPRIS cohort.[12] From the Netherlands, the Lifelines COVID-19 Cohort (*N* = 61,240) was analysed.[13] From the UK, the University College London (UCL) COVID-19 Social Study (*N* = 70,538) was analysed. The total number of unique respondents in our study was 205,084. The DNBC, the UCL COVID-19 Social Study, Lifelines, Constances, and TEMPO each had longitudinal data collections between March – July 2020. The Citizen Science cohort consists of an online survey, completed primarily during a four-day window at the end of March 2020. The Epinion study had a distinct design with independent samples that were surveyed at each of the 20 time-points during the study period. Response- and retention rates for all cohorts are described in detail in Supplemental Table 1.

All survey respondents in all cohorts gave informed consent, and all studies were approved by the appropriate review boards as described in Supplemental Text 1.

### 2.2 Publicly available data sources

Dates of key events, including societal lockdowns, political announcements, and the easing of restrictions, were extracted from national news outlets and Wikipedia. Data on daily hospitalisations and death rates due to COVID-19 were extracted from the websites of the Statens Serum Institut in Denmark, the Ministry for Solidarity and Health (Ministère des Solidarités et de la Santé) in France, the National Institute for Public Health and the Environment (Rijksinstituut voor Volksgezondheid en Milieu) in the Netherlands, and the Government of the UK.

### 2.3 Outcomes and other variables

A detailed description of all variables and outcomes from each cohort survey used in this study is presented in Supplemental Table 2. In brief, considering the main outcomes, the level of worries was ascertained by asking the question “How worried are you about the COVID-19 crisis?” Responses were collected on a 1–10 Likert scale for most cohorts (1–5 for TEMPO, but responses were transformed to a 1–10 scale to facilitate direct comparisons). In separate questions, individuals were asked to respond with “Yes” or “No” to specific worries, precautionary behaviors (Citizen Science, DNBC, Epinion, TEMPO, Lifelines), and personal experiences (UCL COVID-19 Social Study) during the COVID-19 pandemic. The level of anxiety was assessed using the GAD-7 scale for the UCL COVID-19 Social Study. The level of loneliness was assessed using the UCLA Loneliness Scale (short three-item T-ILS version) for all of the cohorts[14] except for Constances. Responses (1–3 Likert scale) to the three loneliness questions were summed up, with resulting scores ranging from 3–9 for all individuals. This score was dichotomised to high levels of loneliness (≥7) and lower levels of loneliness (<7).[15] In the Constances cohort, loneliness was ascertained by responses to the statement “I felt lonely”, collected on a 1–4 Likert scale. This scale was dichotomised to high levels of loneliness (≥3) and lower levels of loneliness (<3).

### 2.4 Statistical analysis

Statistical analysis was performed using R for the Danish cohorts, STATA for the UCL COVID-19 Social Study, SPSS and R for Lifelines, and SAS for the French cohorts.

National census data from the four participating countries were used to weight individuals from general-population cohorts in order to achieve more representative sample sets for statistical analyses (see detailed descriptions of these methods in Supplemental Text 2). In brief, weighting was performed using the raking method [16] for Epinion, Citizen Science and Lifelines, the entropy balancing method [17] for the UCL COVID-19 Social Study, and marginal calibration weighting for Constances.[18] No weighting was performed for DNBC and TEMPO, as these cohorts were not established to be representative samples of the general population. Comparisons between responders and non-responders were possible in three cohorts: Constances, TEMPO, and Lifelines. These comparisons are described in detail in Supplemental Text 3.

We assumed a missing-not-at-random (MNAR) pattern for mental health outcomes, but due to the complexities of imputing multivariate time-series/prospective data, and our assumption that the observed low rates of missing values (0–11% in all analytic steps) will not meaningfully impact our main results, we opted for complete-case analyses, and the appropriate discussion of potential biases and limitations of this approach in our critical appraisal of the results. Thus, in all analytic steps, only survey responses with available outcomes (for the particular analytic step) were analysed and presented, and we report missingness rates (as defined as number of missing responses / all survey respondents) for each part of the analysis.

For the outcome related to level of worries, (weighted or unweighted) means and standard deviations were calculated for each timepoint. For the other outcomes, we calculated the (weighted or unweighted) proportions of individuals who responded “Yes” to survey questions, and of individuals with high levels of anxiety/loneliness.

Proportions of high level of loneliness were calculated in the overall populations and in population strata defined by gender (women and men), age (under 30 years, between 30–60 years, and above 60 years), educational attainment (low, medium, and high level of education) and previous diagnoses of chronic disease (yes/no), and mental illness (yes/no).

### 2.5 Role of the funding source

The funders had no role in the study design, data collection, data analysis, interpretation, the writing of the report or decisions on where to publish.

## 3 Results

Descriptives for all cohorts are reported in Supplemental Table 3.

### 3.1 Country-wide pandemic and intervention timelines

**Fig. 2:**
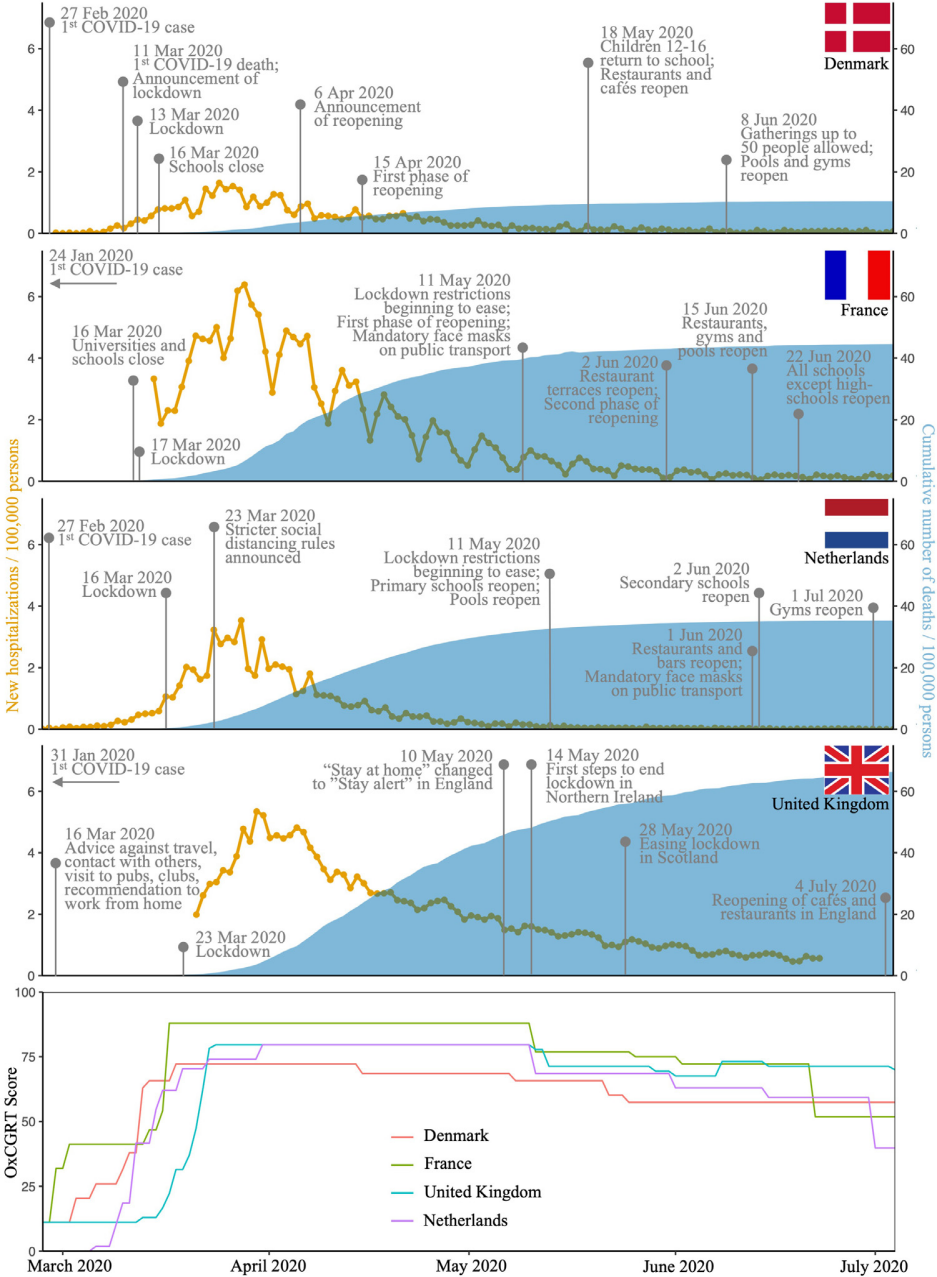
Governmental intervention timelines for Denmark, France, the Netherlands and the United Kingdom. New hospitalisations / 100,000 population (orange) and cumulative number of deaths / 100,000 population (blue) are presented for Denmark (28 Feb 2020 - 04 Jul 2020), France (28 Feb 2020 - 03 Jul 2020), the Netherlands (27 Feb 2020 - 30 Jun 2020) and the United Kingdom (28 Feb 2020 - 04 Jul 2020). The Oxford COVID-19 Government Response Tracker (OxCGRT) Score [0-100] is presented below the governmental intervention timelines (27 Feb 2020 - 04 Jul 2020).

Fig. 2 presents the country-wide timelines of governmental interventions. They highlight main events, such as the initial societal lockdowns, important announcements, the easing of restrictions, and the number of COVID-19-related new hospitalisations and cumulative number of deaths between March–July 2020. Lockdowns were instituted in all four countries, starting on 13 March 2020 in Denmark, 16 March in the Netherlands, 17 March in France, and 23 March in the UK. Alongside the national governmental intervention timelines, we also present the *Oxford COVID-19: Government Response Tracker*, [19] a score [0–100] that ranks a range of governmental policies in response to the pandemic. A gradual easing of the societal lockdowns was observed in all four countries: Denmark started the first phase of its reopening on 15 April, the UK on 10 May, and the Netherlands and France both on 11 May. By early July 2020, the total number of deaths had risen to 606 in Denmark, 6,101 in the Netherlands, 29,893 in France, and 44,198 in the UK. When adjusting for population size and comparing deaths/100,000 individuals, these numbers correlate to 10·44 for Denmark, 35·31 for the Netherlands, 44·62 for France, and 66·31 for the UK.

### 3.2 Worries, anxiety, and precautionary behaviours

**Fig. 3:**
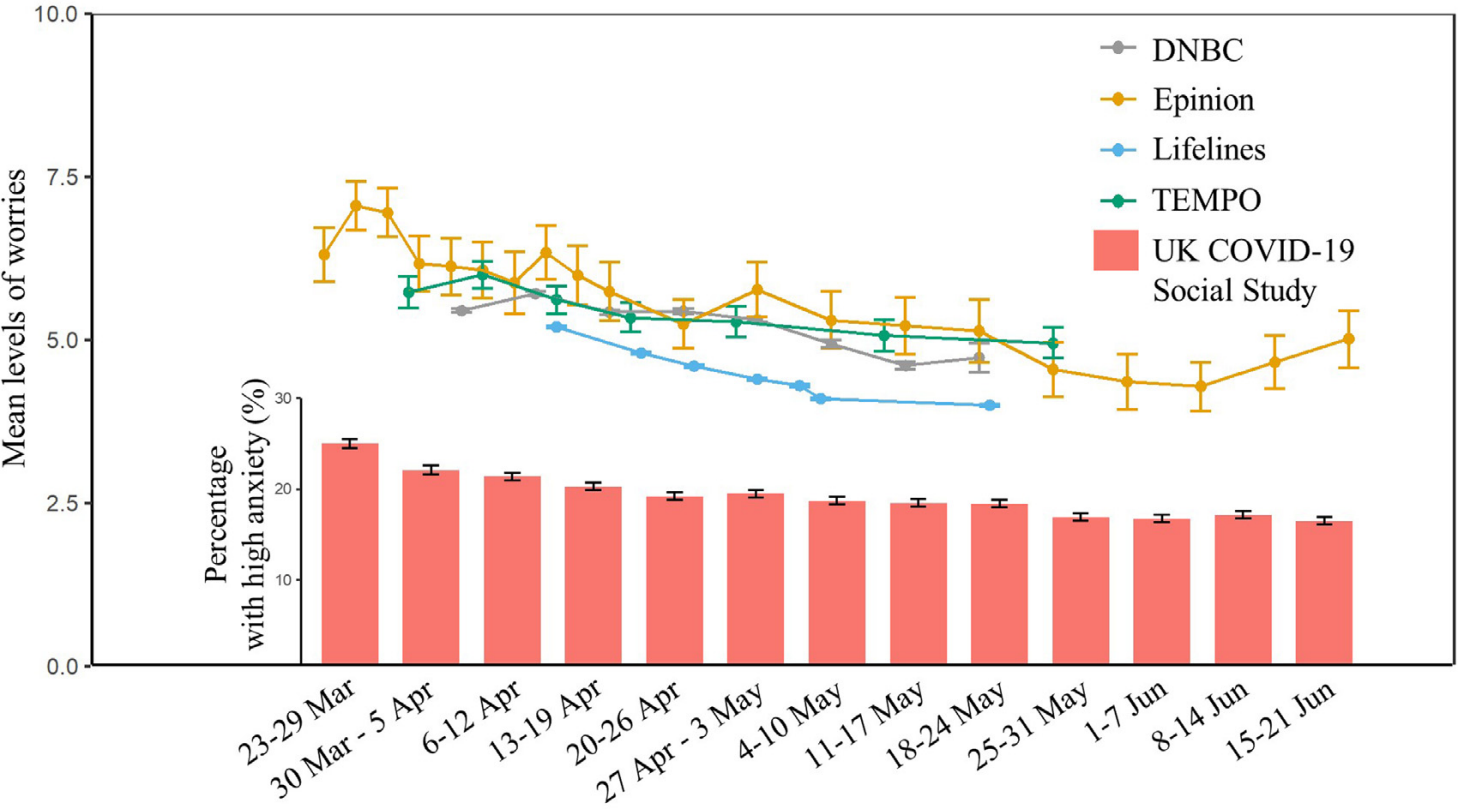
Worries and anxiety about the COVID-19 pandemic in Denmark, France, the Netherlands and the United Kingdom (N=140,495). The graph presents weighted means and 95% CIs of levels of worries in individuals from the Epinion general population cohort (N_total_=2,123) and the Lifelines cohort (N_total_=44,076), and unweighted means and 95% CIs of levels of worries in individuals from the DNBC cohort (N_total_=23,029) and the TEMPO cohort (N_total_=729). On the same graph, weighted proportions are presented of individuals reporting high levels of anxiety in the UCL COVID-19 Social Study (N_total_=70,538).

Fig. 3 shows the results from the time-series data on worries or anxiety related to the COVID-19 pandemic and societal lockdowns. The landscape of worries and anxiety in each country suggests poor mental-health outcomes in the beginning of the pandemic in all four countries, although lower baseline levels of worries were observed in the Netherlands compared to other countries. In the UK, for instance, the highest prevalence of anxiety (25%) was reported at the end of March 2020. After this initial spike in mental-health outcomes, there was a steady decrease in worries or anxiety in all four countries, although the trend in France decreased only very slowly over time. Amongst all responders who were considered for this part of the analysis, the French sample had 0–3%, and the Dutch sample had 11% missingness rates throughout the data collection period. The Danish, and UK samples had no missing data.

**Fig. 4:**
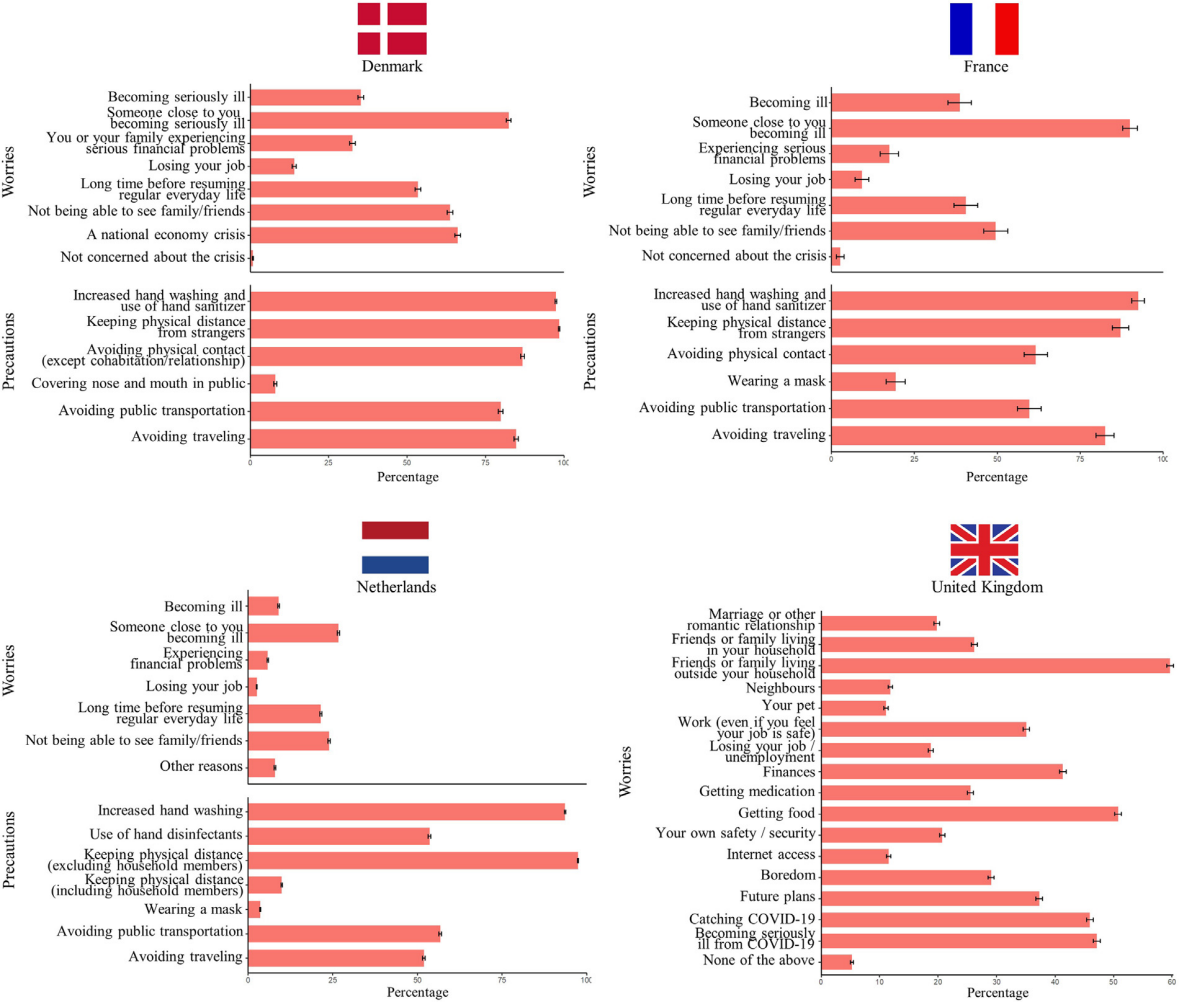
Specific worries and precautions in response to the COVID-19 pandemic in Denmark, France, the Netherlands and the United Kingdom (N=99,840). Weighted proportions of individuals answering “Yes” to questions about specific worries and precautions during the corona crisis in the Citizen Science cohort (N=11,494), the Lifelines cohort (N=59,387) and the UCL COVID-19 Social Study (N=28,230), and unweighted proportions of individuals answering “Yes” to questions about specific worries and precautions during the corona crisis in the TEMPO cohort (N=729).

Fig. 4 presents statistics for specific worries and precautions during the societal lockdowns. Approximately 40% of the population in each country (except in the Netherlands, where the numbers were lower) was concerned about becoming seriously ill during the first months of the pandemic. Even more people were worried about someone close to them becoming ill, ranging from 90% in France to 27% in the Netherlands. Only a very small fraction of each sample (<5%) reported not being at all worried about the pandemic.

Apart from wearing a face-mask, for which there were different rules and commendations in each country, the majority of the respondents in all four countries reported complying with the recommended precautions and preventive measures, including increased handwashing, using hand sanitiser, physical distancing, avoiding physical contact outside of the household, and avoiding public transportation and other forms of travel (face-mask reported by <25% in all countries; all other precautions reported by >50% in all countries). Amongst all responders who were considered for this part of the analysis, the Dutch sample had a 3% missingness rate. The Danish, French, and UK samples had no missing data.

### 3.3 Loneliness

**Fig. 5:**
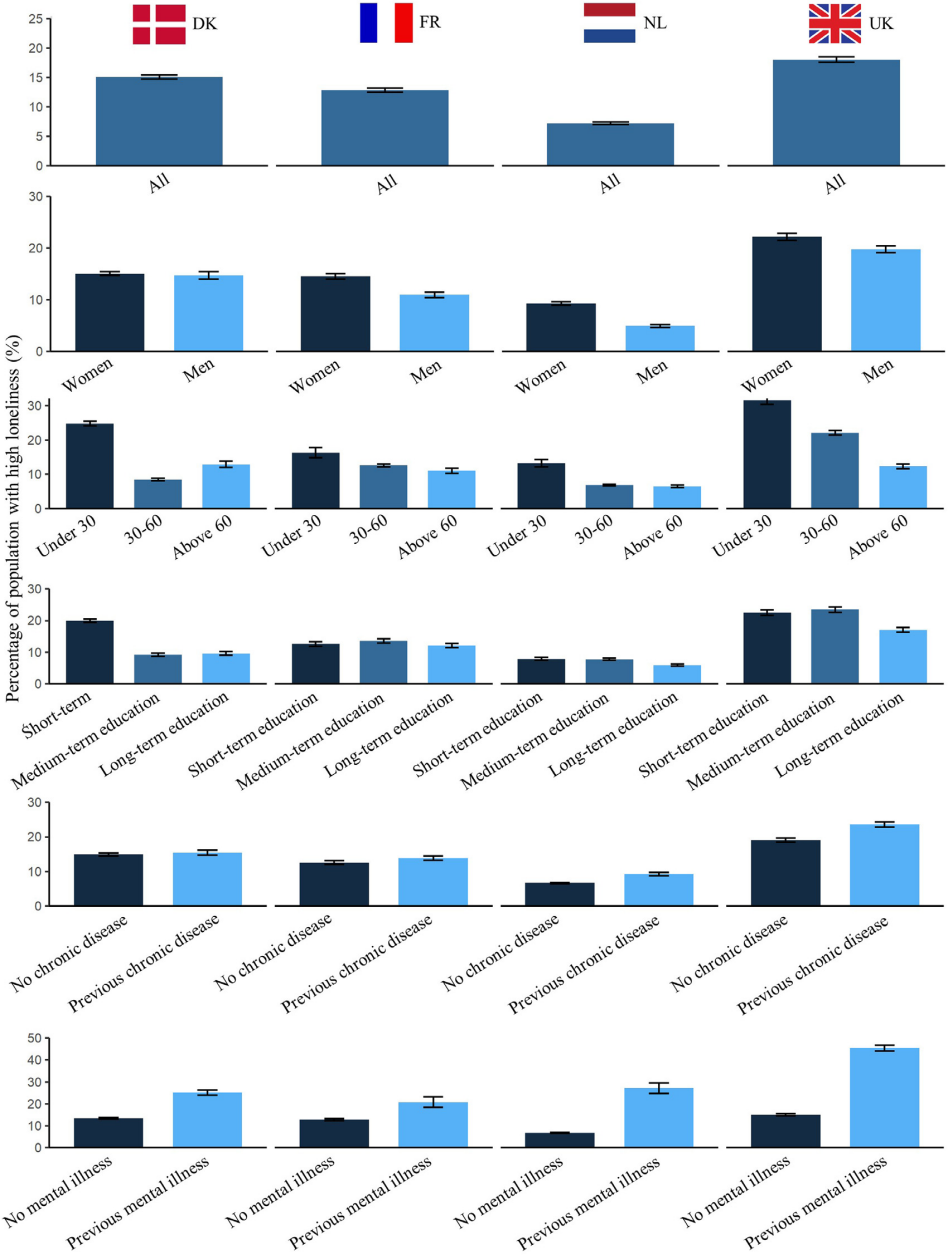
Loneliness during the COVID-19 pandemic in Denmark, France, the Netherlands and the United Kingdom (N=158,692). Weighted proportions of individuals with high levels of loneliness in the Constances cohort (N=29,974), the Lifelines cohort (N=57,885) and the UCL COVID-19 Social Study (N=28,230), and unweighted proportions of individuals reporting high levels of loneliness in the combined set of Danish citizens from the DNBC cohort (N=24,724), the Citizen Science cohort (N=11,494) and the Epinion cohort (N=6,385).

Fig. 5 shows the proportion of individuals who reported high loneliness within the overall population and various subgroups defined by age, gender, educational status, and history of chronic and/or mental illness. Loneliness in each cohort was ascertained in the beginning of the lockdown. Across all four countries, the highest levels of loneliness were reported by those younger than 30 years of age (24·8%,16·3%,13·2%, and 31·6% for Denmark, France, the Netherlands, and the UK, respectively) as well as those reporting previously diagnosed mental illness (25·2%,20·8%,27·2%, and 45·4% for Denmark, France, the Netherlands, and the UK, respectively). Slightly higher levels of loneliness were observed amongst women, and individuals with previously diagnosed chronic diseases. Amongst all responders who were considered for this part of the analysis, the Danish sample had 5%, the French sample had 3%, and the Dutch sample had 5% missingness rates. The UK sample had no missing data.

## 4 Discussion

In March 2020, many countries around the world implemented strict public-health measures to mitigate the spread of the SARS-CoV-2 virus. Previous research indicates that such measures can lead to psychological distress, which has the potential to develop into long-term and severe mental illness.[20] Here we analysed prospective, time-series cohort data from four Western and Northern European countries in order to assess mental-health outcomes, such as levels of loneliness, worries, anxiety, and precautionary behaviours in response to the pandemic. Using a synchronised survey to assess these outcomes, our investigation aimed to reveal differences and similarities between governmental interventions. Loneliness, in particular, is a prominent risk factor for future anxiety and depression; thus, we also aimed to investigate whether certain subgroups of the population samples demonstrated poorer mental-health outcomes compared to others. One of the main aims of this study was also to identify groups that might have benefitted more from targeted public-health interventions during the initial months of the COVID-19 pandemic and/or in future crises. Recent results from the UK Biobank suggest that higher levels of psychosocial distress and neuroticism are associated with higher levels of COVID-19-related hospitalisations; [21] thus, we believe that healthcare systems would benefit from identifying subgroups of individuals who are more susceptible to anxiety and loneliness during the physical-distancing phases of lockdowns in order to reduce downstream healthcare burdens.

The most striking difference between the four countries in our study is that levels of worries and loneliness, and the proportion of individuals with specific worries and precautionary behaviours, were all lower in the Netherlands compared to the other countries. This trend cannot be explained by the timing of the national public-health interventions, which were comparable between countries, nor by the level of stringency of the Dutch government during the crisis (the second-most stringent amongst the four countries analysed for the main lockdown period, April 2020). A possible explanation for this systematic difference is that the Lifelines cohort recruited individuals from the northern part of the Netherlands, where COVID-19 infection rates were much lower compared to national levels. This was possibly due to the delayed arrival of the virus compared to the southern part of the country, the lack of large-scale gatherings, better testing infrastructures and that there are fewer densely populated areas in the northern part of the Netherlands.[13] Other reasons for better mental-health outcomes in the Lifelines cohort could also be the Netherlands’ ‘intelligent lockdown’ strategy combined with high trust in the government; such factors might have helped the population maintain lower levels of worries and loneliness during the first months of the crisis.

Despite this specific trend, our overall results from the four countries indicate more similarities than differences: The general state of worries was high at the beginning of the pandemic and tended to decrease steadily throughout the subsequent months; specific governmental announcements and interventions did not seem to result in sudden changes in levels of experienced worries or anxiety in any of the populations. The landscape of specific worries indicates that, while a high proportion of individuals were worried about becoming ill, they were even more worried about loved ones becoming ill. This finding was previously observed elsewhere in a smaller survey study of ~400 adults from the UK.[22] In our study, high levels of loneliness were observed in younger people (<30 years old) and individuals with previous diagnoses of mental illness across all four countries. Slightly higher levels of loneliness were also observed in people diagnosed with a chronic illness as well as women in all four countries. We compared levels of loneliness in our study to levels prior to March 2020 in the UK; [23] our search demonstrated higher levels of loneliness during the first months of the COVID-19 pandemic compared to previous years.

Some of the subgroup trends in our study, namely that younger individuals, women and those with pre-existing chronic conditions experience higher levels of loneliness, have been observed elsewhere in smaller samples.[24], [25], [26] However, to date, only studies with much smaller sample sizes point to such high levels of loneliness in individuals with a history of mental illness.[27,28] Our results indicate that 20–50% of those with a history of mental illness experienced high levels of loneliness during the first months of lockdown. This is an important, robust, and novel finding because it identifies the specific subgroup that, along with younger individuals, might benefit the most from tailored interventions designed to alleviate loneliness and prevent more serious, long-term health consequences.

We hypothesise that younger individuals who reported being more lonely and socially isolated before the pandemic [29] and individuals with a history of mental illness may have experienced poor mental-health outcomes during the first months of the pandemic for different reasons. Possible reasons include a general fear of illness and the SARS-CoV-2 virus itself, as well as the governmental authorities’ communication strategies. But they could also arise from the preventive measures themselves, such as physical distancing, and restricted freedom of movement. Nevertheless, it is likely that these high-risk groups would benefit from tailored strategies to help them cope with such crises. For people with a history of mental illness, strategies might include digital interventions, to alleviate stress, uncertainty, and other concerns. Younger individuals might benefit from fewer restrictions in movement, given that they are generally less likely to become seriously ill from the SARS-CoV-2 virus.[30]

This study has several strengths. First, a large, multinational sample was collected; we report on >200,000 individuals from seven cohorts from four European countries. Second, multiple studies had time-series data available from as many as 20 sampling points during the crisis. It is important to acknowledge the diversity of the included cohorts both from a study design (bulk collection, time-series design, prospective independent samplings) and sample size perspective. While the smaller cohorts generally had more time-points allowing us to track the progression of mental health outcomes, the larger studies enabled us to draw more precise estimates of the mental health of general populations. Third, a harmonised survey made it possible to compare various cohorts across different countries. Fourth, standardised and validated survey items, such as the UCLA Loneliness Scale and the GAD-7 Anxiety Scale, were used to assess key mental-health outcomes. Fifth, the visualisations present a detailed landscape of mental-health outcomes and intervention timelines for all four participating countries; these images allow for comparisons between countries. However, we advise caution when comparing key COVID-19-related metrics, such as the number of hospitalised individuals and deaths between countries; it is possible that countries used different methods to ascertain COVID-19-related hospitalisations and deaths. For instance, prior to the end of March 2020, COVID-19-related deaths in France were only reported in hospitals, whereas it is not apparent from other countries’ official statistics whether or not the number of deaths in March was only collected in hospitals. Moreover, while the number of newly hospitalised individuals follows a weekly pattern in official French data, no such patterns are immediately apparent in the other countries. Such inconsistencies make it difficult to compare these metrics between countries.

An important limitation of this study is that the general-population samples are not entirely representative of the national populations. Such sampling bias is a common phenomenon in survey studies, and we aimed to circumvent this limitation by applying various techniques to weight these samples so that underrepresented subgroups gained more weight in analyses. Of note, some included cohorts are not representative of general populations by design (the DNBC is a birth cohort of mothers and their offspring, and the TEMPO cohort includes employees of the French national gas and electricity firms), and we did not apply weighting to these cohorts. We recommend that the presented data should be interpreted mostly in light of the longitudinal patterns they show. For accurate prevalence rates for mental ill-health, representative population cohorts are needed. All analysed data were self-reported and thus prone to various biases associated with this type of data collection. The two most important are *response bias* (systematic error between responses and true values) and *nonresponse bias* (differences in true values between responders and non-responders). While mental-health outcomes are generally prone to underreporting, it is difficult to speculate whether such biased reporting exists in our study's surveys, as the surveys were largely focused on psychosocial wellbeing during the COVID-19 crisis. We find it unlikely that response bias would significantly alter our findings. In most cohorts, an overarching theme is that the individuals who completed the surveys are generally more likely to be older, to be women and have a higher educational attainment compared to non-responders. To alleviate nonresponse bias, we presented age-, gender-, education-, and disease history-stratified results for our key outcome, loneliness. Our data had low rates of missingness. However, we assume that those who opted not to respond to specific questions about mental health might systematically differ from those who chose to respond. Namely, it is likely that non-responders to these questions have worse outcomes compared to responders, thus biasing our observed results towards a more optimistic overview of mental health landscapes.

Last, while these are the first international comparative results on mental health outcomes during the COVID-19 pandemic, we acknowledge that the findings are limited to four high-income Western and Northern European countries and the specific cohorts analysed. Our collaboration was initiated at the very beginning of the pandemic, which ensured comparable measures across several cohorts, but future work should include a more systematic assessment of mental health outcomes across both high- and low-income countries, from various regions of the world. This is especially important as it is likely that various populations have different perceptions of public health authorities, attitudes towards governments, and possess different core ethical, moral, and cultural values, and these features might play a key role in how mental health outcomes are shaping in response to governmental interventions.

In conclusion, our study indicates that younger individuals and people with a history of mental illness experienced higher levels of loneliness compared to other subgroups during the first four months of the societal lockdowns related to the COVID-19 pandemic. As the pandemic continues, future governmental measures should include targeted strategies for these subgroups in order to decrease the risk of serious long-term health consequences. As noted above, one size might not fit all: it is important to devise strategies that are effective for the right groups, at the right time. Well-designed digital tools can offer such tailored solutions by considering the needs of the target populations. For instance, phone pals or collaborative games could be effective in alleviating stress and loneliness in individuals with a history of mental illness, while younger individuals could possibly benefit from fewer restrictions. In designing these targeted interventions, it is also important to consider factors that might differ between countries (e.g. elderly populations might have varying attitudes towards digital solutions across countries). Our collaborative study is the first to show consistency between mental-health landscapes and subgroup trends across four European countries, despite their varying governmental interventions, lockdown strategies, and general stringency. We believe that a coordinated international strategy and increased collaboration would be effective steps in counteracting the negative impacts of the pandemic and its related lockdowns on mental health.

## 5 Contributions

The corresponding authors attest that all listed authors meet the ICMJE authorship criteria and that no others meeting the criteria have been omitted. TVV and NHR are the guarantors of this manuscript. TVV, AC, and NHR wrote the first draft of the manuscript. TVV, DF, and NHR were responsible for the conceptualization of the project. TVV, BF, ASD, LKE, JJHB, JM, SKRvZ, KH, KS, AC, and NHR curated the data. TVV, BF, ASD, LKE, JJHB, JM, and SKRvZ analysed the data. TVV generated all visualisations. SB, UB, DF, KH, MG, MM, KS, MZ, and NHR acquired funding and provided supervision. All listed authors reviewed and edited the manuscript and approved the final, submitted version. TVV, FB, JJHB, JM, SKRvZ, SB, UB, DF, MG, MM, KS, MZ and NHR verified the underlying data. All authors confirm that they had full access to the data in the study and accept responsibility to submit for publication.

## Declaration of Competing Interests

All authors have completed the ICMJE uniform disclosure form at www.icmje.org/coi_disclosure.pdf and declare: no support from any organisation for the submitted work; no financial relationships with any organisations that might have an interest in the submitted work in the previous three years; no other relationships or activities that could appear to have influenced the submitted work.

## Data sharing statement

Due to ethical reasons, the PIs of the participating cohorts are not able to share individual-level data. Regarding potential opportunities for collaboration, please contact the corresponding authors.

## Funding

The data collection related to the project ‘Standing together – at a distance: how Danes are living with the corona crisis’ was supported by the VELUX FOUNDATIONS’ special pool for data-collection projects related to studying COVID-19, grant no. 36336.

The Danish National Birth Cohort was established with a significant grant from the Danish National Research Foundation. Additional support was obtained from the Danish Regional Committees, the Pharmacy Foundation, the Egmont Foundation, the March of Dimes Birth Defects Foundation, the Health Foundation and other minor grants. The DNBC Biobank has been supported by the Novo Nordisk Foundation and the Lundbeck Foundation. Follow-up of mothers and children have been supported by the Danish Medical Research Council (SSVF 0646, 271-08-0839/06-066023, O602-01042B, 0602-02738B), the Lundbeck Foundation (195/04, R100-A9193), The Innovation Fund Denmark 0603-00294B (09-067124), the Nordea Foundation (02-2013-2014), Aarhus Ideas (AU R9-A959-13-S804), University of Copenhagen Strategic Grant (IFSV 2012), and the Danish Council for Independent Research (DFF – 4183-00594 and DFF - 4183-00152). LKE was supported by a Rubicon grant (45219105) of the Netherlands Organisation for Health Research and Development (ZonMw). TVV is supported by the Department of Public Health (University of Copenhagen).

The CONSTANCES COVID-19 Study was funded by: ANR (Agence Nationale de la Recherche, #ANR-20-COVI-000, #ANR-10-COHO-06), Fondation pour la Recherche Médicale (#20RR052-00), Inserm (Institut National de la Santé et de la Recherche Médicale, #C20-26). The CONSTANCES cohort is supported by the Caisse Nationale d'Assurance Maladie (CNAM), the French Ministry of Health, the Ministry of Research, and the Institut national de la santé et de la recherche médicale. CONSTANCES benefits from a grant from the French National Research Agency [grant number ANR-11-INBS-0002] and is also partly funded by MSD, AstraZeneca, Lundbeck and L'Oréal.

The TEMPO cohort received funding from the French National Research Agency (ANR: Programme Jeune Chercheur; Flash COVID-19); the French Institute for Public Health Research-IReSP (TGIR Cohortes); the French Inter-departmental Mission for the Fight against Drugs and Drug Addiction (MILDeCA); the French Institute of Cancer (INCa); and the Pfizer Foundation.

The Lifelines initiative has been made possible by subsidy from the Dutch Ministry of Health, Welfare and Sport, the Dutch Ministry of Economic Affairs, the University Medical Center Groningen (UMCG), Groningen University and the Provinces in the North of the Netherlands (Drenthe, Friesland, Groningen).

The UCL Covid-19 Social Study was funded by the Nuffield Foundation [WEL/FR-000022583], but the views expressed are those of the authors and not necessarily the Foundation. The study was also supported by the MARCH Mental Health Network funded by the Cross-Disciplinary Mental Health Network Plus initiative supported by UK Research and Innovation [ES/S002588/1], and by the Wellcome Trust [221400/Z/20/Z]. DF was funded by the Wellcome Trust [205407/Z/16/Z].

The funders had no role in the study design, data collection, data analysis, interpretation, the writing of the report or decisions on where to publish.

## Appendix Supplementary materials

Supplemental Text 1. Cohort Descriptions and Ethical Considerations Image, application 1

Supplemental Text 2. Weighting methods Image, application 2

Supplemental Text 3. Comparisons of responders and non-responders Image, application 3

Supplemental Table 1. Response and retention rates for all cohorts. Image, application 4

Supplemental Table 2. Variable descriptions Image, application 5

Supplemental Table 3. Cohort descriptives Image, application 6

## Footnotes

Supplementary material associated with this article can be found, in the online version, at doi:10.1016/j.lanepe.2020.100020.
