# Supplementary material for: Loneliness, worries, anxiety, and precautionary behaviours in response to the COVID-19 pandemic: A longitudinal analysis of 200,000 Western and Northern Europeans
Source: Lancet Reg Health Eur. 2021 Jan 2;2:100020. doi: 10.1016/j.lanepe.2020.100020 (PMC8042675; doi:10.1016/j.lanepe.2020.100020)
Supplement: Supplementary file 1 — Supplemental Text 1. Cohort Descriptions and Ethical Considerations [file mmc1.docx]

**Supplemental Text 1. Cohort Descriptions and Ethical Considerations**

**Citizen Science (DK)**

An interdisciplinary research project ‘Standing together – at a distance: how Danes are living with the corona crisis’ was established in early March 2020, and a series of timed and harmonized online surveys were initiated on the 20^th^ March to document the immediate effects of the Danish lockdown on mental health amongst different population groups. The website https://coronaminds.ku.dk/english/ was established to collect data from the public and engage them. The recruitment to the Citizen Science sample was undertaken in collaboration with the Danish newspaper Politiken. The total number of individuals used from the Citizen Science cohort is 11,494.

*Ethical considerations: Ethical and personal data handling approval was obtained from the Department of Public Health at the University of Copenhagen (514-0485/20-3000). All survey respondents agreed to participate in the study and gave informed consent. All data is process according to the Personal Data Ordinance, Regulation 2016/679.*

**Danish National Birth Cohort (DK)**

An interdisciplinary research project ‘Standing together – at a distance: how Danes are living with the corona crisis’ was established in early March 2020, and a series of timed and harmonized online surveys were initiated on the 20^th^ March 2020 to document the immediate effects of the Danish lockdown on mental health amongst different population groups. The online surveying was initiated in the DNBC between the 30^th^ March 2020 and 2^nd^ April 2020. Participants with an available email address / telephone number were invited. From the outset, 53,323 adolescents (aged 16–24) and 53,968 mothers were invited to participate. Data were available for 13,002 adolescents and 14,075 mothers at baseline. Respondents who completed the first questionnaire within one week were subsequently invited to participate in weekly surveys until the 14^th^ May 2020 (and subsequently, if they responded to the second questionnaire, they were invited for the third questionnaire, etc.).

*Ethical considerations: The DNBC is approved by the Danish Data Protection Agency (18/04608) and the Committee on Health Research Ethics (case no. (KF) 01-471/94). The DNBC participants were enrolled by informed consent. Ethical and personal data handling approval was obtained from the Department of Public Health at the University of Copenhagen (514-0497/20-3000). All survey respondents agreed to participate in the study and gave informed consent. 107.291*

**Epinion (DK)**

An interdisciplinary research project ‘Standing together – at a distance: how Danes are living with the corona crisis’ was established in early March 2020, and a series of timed and harmonized online surveys were initiated on the 20^th^ March 2020 to document the immediate effects of the Danish lockdown on mental health amongst different population groups. A time series (20 time-points, so far) of cross-sectional, online surveys were sent out to three times ~100 Danish residents drawn from the three population groups: 1. elderly people aged >65 years, 2. families with children living at home, and 3. a sample of the general population aged 18–89. This data collection was undertaken in collaboration with the consumer-research company Epinion. Starting on the 20^th^ March 2020, the data collection was conducted every three days, and from the 16^th^ April, the data collection continued to occur once a week. The total, combined samples size from the Epinion cohort is 6,385.

*Ethical considerations: Ethical and personal data handling approval was obtained from the Department of Public Health at the University of Copenhagen (514-0485/20-3000). All survey respondents agreed to participate in the study and gave informed consent. All data is process according to the Personal Data Ordinance, Regulation 2016/679.*

**Constances (FR)**

Constances is a large, population-based, prospective cohort whose recruitment began in 2012 and ended in 2019 with a total size of more than 200,000 subjects, including volunteers aged 18 to 69 years at baseline and living in 21 selected departments (administrative divisions) throughout metropolitan France, in both rural and urban settings, affiliated to the social security system. A questionnaire was sent to all individuals participating in the online cohort during lockdown (approximately 66,840 individuals). Chronic diseases were collected using an annual self-administered follow-up questionnaire completed by participants at home, using either a paper questionnaire or internet. The total number of unique respondents used in this study was 29,974.

*Ethical considerations: The Constances cohort study has received the authorization of the French Data Protection Authority (CNIL: Commission Nationale de l’Informatique et des Libertés) and the institutional review board of the National Institute for Medical Research (Authorization number 910486). Ethical approval and written or electronic informed consent for participation in the SAPRIS cohort were obtained from each participant before enrolment in the original cohort. The SAPRIS survey was approved by the Inserm ethics committee (approval #20-672 on March 30, 2020). All subjects included in this study gave their informed consent.*

**TEMPO (FR)**

The TEMPO study is a French ongoing prospective cohort which aims to evaluate individual, familial and social determinants of mental social health and addictive behaviors. The initial cohort was established in 1991, recruiting its participants via their parents who participated in the GAZEL cohort study. A second recruitment phase took place in 2011 where all young adults whose parents participated on the GAZEL cohort study were invited. Data collection took place in 1991 (n=2,658) and 1999 (n=1,270). Parents were asked to complete a questionnaire regarding the living conditions and mental health of the participant, and the participants answered the questionnaire themselves in 1999 (n=1,148), 2009 (n=1,103), 2011 (n=1,214), 2014 (n=786), 2018 (n=864) and 2020 (8 questionnaires proposed in total). The data collection in 2020 was undertaken through a web questionnaire, having in consideration the conditions linked to the lockdown due to the COVID-19 pandemic. As of this project, 729 people have participated in the latest phase of data collection.

*Ethical considerations: The TEMPO cohort was approved by the French national committee for data protection (CNIL: Commission Nationale de l’Informatique et des Libertés) (Authorization number 908163). All subjects included in this study gave their informed consent.*

**Lifelines (NL)**

Lifelines is a multi-disciplinary prospective population-based cohort study examining in a unique three-generation design the health and health-related behaviours of 167,729 persons living in the North of the Netherlands. Lifelines employs a broad range of investigative procedures in assessing the biomedical, socio-demographic, behavioural, physical and psychological factors which contribute to the health and disease of the general population, with a special focus on multi-morbidity and complex genetics. Participants for the Lifelines COVID-19 cohort were recruited from the eligible participants of Lifelines and the Lifelines NEXT birth cohort. The aim of the Lifelines COVID-19 cohort is to assess the psychological and societal impacts of the COVID-19 pandemic and to investigate potential risk factors for COVID-19 within the Lifelines prospective population cohort. Digital questionnaires were sent out to all adult Lifelines participants with a known e-mail address, and were valid for three weeks. The first and second questionnaires contain baseline questions to obtain important basic characteristics of all respondents. All other follow-up questionnaires contain a basic set of questions, complemented with a set of additional questions. For the current study, we included all participants that entered the Lifelines COVID-19 cohort during the first or second wave (n=61,240). Participants who entered the cohort from wave three onwards were not included, because information on important baseline characteristics was lacking.

*Ethical considerations: The Lifelines (ethics number: 2007/152) and the Lifelines NEXT (ethics number: 2015/600) studies were approved by the ethics committee of the University Medical Center Groningen. All Lifelines and Lifelines NEXT participants have provided informed consent that provides the opportunity for add-on research.*

**UCL COVID-19 Social Study (UK)**

The COVID-19 Social Study commenced on 21^st^ March 2020 in response to the outbreak of the novel coronavirus disease (COVID-19). It is a large scale longitudinal panel study of adults aged 18 and over living in the UK. Participants are followed weekly until the end of June, 2020. The project website can be found at: https://www.covidsocialstudy.org/. The total sample size used in this project was 70,538.

*Ethical considerations: Ethical approval for the COVID-19 Social Study was granted by the UCL Ethics Committee. All participants provided fully informed consent. The study is GDPR compliant.*
