# Supplementary material for: Loneliness, worries, anxiety, and precautionary behaviours in response to the COVID-19 pandemic: A longitudinal analysis of 200,000 Western and Northern Europeans
Source: Lancet Reg Health Eur. 2021 Jan 2;2:100020. doi: 10.1016/j.lanepe.2020.100020 (PMC8042675; doi:10.1016/j.lanepe.2020.100020)
Supplement: Supplementary file 2 — Supplemental Text 2. Weighting methods [file mmc2.docx]

**Supplemental Text 2. Weighting methods**

**Raking Method – employed in the Citizen Science cohort (DK), the Epinion general population cohort (DK), and the Lifelines cohort (NL)**

The raking method was developed to counteract nonresponse bias and make population samples more representable by up-weighting underrepresented categories. The R package ‘survey’ was used to rake samples using population statistics. The following variables were used to rake the Danish samples: age groups, sex, regions. Population references for 2020 were extracted from Danmarks Statistik [https://statistikbanken.dk/statbank5a/selectvarval/define.asp?PLanguage=0&MainTable=FOLK1A&TabStrip=Select]. The following variables were used to rake Lifelines: age, sex. Population references were extracted from the most recent (2011) Dutch Census data reported by Statistics Netherlands (CBS) [https://www.cbs.nl/en-gb/publication/2014/47/dutch-census-2011].

Reference for method: Dal Grande E, Chittleborough CR, Campostrini S, Tucker G, Taylor AW. Health Estimates Using Survey Raked-Weighting Techniques in an Australian Population Health Surveillance System. *American journal of epidemiology* 2015; **182**(6): 544–56.

**Entropy Balancing Method – employed in the UK COVID-19 Social Study (UK)**

The cross-sectional weighting was implemented by using Stata, using the user-written package ‘ebalance’ [https://web.stanford.edu/~jhain/Paper/JSS2013.pdf]. The weighted data were matched to population statistics for the following domains: age; sex; ethnicity; educational attainment; country of living. These statistics were extracted from the Office for National Statistics

[https://www.ons.gov.uk/peoplepopulationandcommunity/populationandmigration/populationestimates/datasets/populationestimatesforukenglandandwalesscotlandandnorthernireland].

Reference for method: Hainmueller J. Entropy Balancing for Causal Effects: A Multivariate Reweighting Method to Produce Balanced Samples in Observational Studies. *Political Analysis* 2012; **20**(1): 25–46.

**Marginal Calibration Weighting Methods – employed in the Constances cohort (FR)**

The weighting method employed relies on scores estimation that is grouped together as a homogeneous response. This method was applied to calculate the weight of participation to the questionnaire and a weight for participation request to the questionnaire. These two weights allowed for obtaining the final weight. The obtained weights are then calibrated on the margins of the target population of Constances. The calibration variables are the ones that are usually used in the calculation of the annual weightings of Constances. In total, 66,680 individuals were sent a request to participate in the Covid questionnaire (QCovid), out of which 47,326 participated. The target population of Constances included volunteers aged 18 to 69 years affiliated to the social security and living in the 21 selected departments of Constances. The method used is based on the one used to setting up the weightings in the Constances cohort. The variables used are the following: sex, age class, employment status, geographical location, affiliation to the social security.

Reference for method: Deville JC, Särndal CE. Calibration estimators in survey sampling. *Journal of the American Statistical Association* 1992; **87**: 376-82.

**No weighting – employed in the DNBC cohort (DK), and the TEMPO cohort (FR)**
