# Supplementary material for: Loneliness, worries, anxiety, and precautionary behaviours in response to the COVID-19 pandemic: A longitudinal analysis of 200,000 Western and Northern Europeans
Source: Lancet Reg Health Eur. 2021 Jan 2;2:100020. doi: 10.1016/j.lanepe.2020.100020 (PMC8042675; doi:10.1016/j.lanepe.2020.100020)
Supplement: Supplementary file 3 — Supplemental Text 3. Comparisons of responders and non-responders [file mmc3.docx]

**Supplemental Text 3. Comparisons of responders and non-responders**

Responders and non-responders were compared in all cohorts where this was possible. Comparisons were not possible in the Epinion time-series survey (DK), the Citizen Science sample (DK), the Danish National Birth Cohort (DK) and the UCL COVID-19 Social Study (UK) as no information was available on non-responders. We undertook comparisons between responders and non-responders with regards to key sociodemographic factors: age, sex and educational status. We conducted Pearson’s chi-squared tests for comparisons of the two categorical variables (sex, educational status) and independent samples t-tests for comparisons of age.

**Lifelines (NL)**

|  | Responders | Non-responders |
| --- | --- | --- |
| Education (N = 148,805) | N = 60,000 | N = 88,805 |
| Long-term education | 20,949 (35%) | 23,550 (26%) |
| Medium-term education | 23,536 (39%) | 35,291 (40%) |
| Short-term education | 15,515 (26%) | 29,964 (34%) |
| Pearson’s chi squared test | *P* < 2.2x10^-16^ | |
|  |  |  |
| Sex (N = 152,728) | N = 61,240 | N = 91,488 |
| male | 23,950 (39%) | 39,438 (43%) |
| female | 37,290 (61%) | 52,050 (57%) |
| Pearson’s chi squared test | *P* < 2.2x10^-16^ | |
|  |  |  |
| Age (N = 152,728) | N = 61,240 | N = 91,488 |
|  | mean: 46.39 | mean: 43.46 |
|  | SD: 12.225 | SD: 13.579 |
| Independent samples t-test | *P* < 0.0001 | |

**Constances (FR)**

|  | Responders | Non-responders |
| --- | --- | --- |
| Education (N = 51,984) | N = 39,293 | N = 12,691 |
| Long-term education | 13,373 (34.03%) | 3,811 (30.03%) |
| Medium-term education | 15,249 (38.81%) | 4,750 (37.43%) |
| Short-term education | 10,671 (27.16%) | 4,130 (32.54%) |
| Pearson’s chi squared test | *P* < 2.2x10^-16^ | |
|  |  |  |
| Sex (N = 52,556) | N = 39,688 | N = 12,868 |
| male | 19,429 (49%) | 6,597 (51%) |
| female | 20,259 (51%) | 6,271 (49%) |
| Pearson’s chi squared test | *P* = 5.4 x10^-6^ | |
|  |  |  |
| Age (N = 52,556) | N = 39,688 | N = 12,868 |
|  | mean: 48.15 | mean: 42.9 |
|  | SD: 13.1 | SD: 13.15 |
| Independent samples t-test | *P* < 0.0001 | |

**TEMPO (FR)**

|  | Responders | Non-responders |
| --- | --- | --- |
| Education (N = 1,194) | N = 728 | N = 466 |
| Long-term education | 342 (47%) | 159 (34%) |
| Medium-term education | 310 (43%) | 219 (47%) |
| Short-term education | 76 (10%) | 88 (19%) |
| Pearson’s chi squared test | *P* = 1.2x10^-6^ | |
|  |  |  |
| Sex (N = 1,197) | N = 729 | N = 468 |
| male | 257 (35%) | 184 (39%) |
| female | 472 (65%) | 284 (61%) |
| Pearson’s chi squared test | *P* = 0.17 | |
|  |  |  |
| Age (N = 1,197) | N = 729 | N = 468 |
|  | mean: 40.1 | mean: 39.4 |
|  | SD: 3.6 | SD: 3.9 |
| Independent samples t-test | *P* = 0.0015 | |
